# Ranibizumab non-response in pachychoroid neovasculopathy: Effects of switching to aflibercept

**DOI:** 10.1038/s41598-020-65370-w

**Published:** 2020-05-21

**Authors:** Benedikt Schworm, Nikolaus Luft, Leonie F. Keidel, Tina R. Herold, Armin Wolf, Siegfried G. Priglinger, Jakob Siedlecki

**Affiliations:** Department of Ophthalmology, Ludwig-Maximilians-University, Munich, Germany

**Keywords:** Medical research, Outcomes research

## Abstract

Non-response to intravitreal ranibizumab represents a frequent problem in pachychoroid neovasculopathy (PNV). To investigate the effectivity of switching to aflibercept, the database of the Ludwig Maximilians University, Munich, was screened for patients fulfilling the following inclusion criteria: (i) diagnosis of PNV; (ii) inadequate response to ≥ 3 ranibizumab injections, in spite of monthly dosing, defined as persistence of subretinal-fluid four weeks after the last ranibizumab injection; (iii) resulting switch to aflibercept administered as three monthly injections. Primary outcome measure was percentage of eyes with a dry macula four weeks after the third aflibercept injection. Secondary outcome measures included changes in maximum subretinal fluid (SRF), central subfield thickness (CST) and subfoveal choroidal thickness (SFCT). In total, 14 eyes of 14 patients were included. Mean age was 64.1 ± 7.5 (range: 51–78) years. Switching to aflibercept was performed after mean 8.4 ± 4.1 (3–15) ranibizumab injections. While no eye (0%) achieved a dry macula status during ranibizumab treatment, switching to aflibercept achieved a dry macula status in eight eyes (57.1%) after three injections. While both ranibizumab and aflibercept showed an effect on CST (p = 0.027, p = 0.003), only aflibercept showed a significant effect on SRF (p = 0.0009) and SFCT (p = 0.044). In cases of PNV not responding to intravitreal ranibizumab, switching treatment to aflibercept induces a favorable short-term response resolving persistent fluid and achieving a dry macula. Further studies with longer follow-up are warranted.

## Introduction

Chronic pachychoroid disorders can frequently lead to the development of a choroidal neovascularization (CNV)^1–3^. Recently, both Hwang *et al*.^4^ and we^5^ have noted a significant overlap between the two entities of *central serous chorioretinopathy* (CSC) *complicated by CNV* and *pachychoroid neovasculopathy (PNV)*. For this reason, and due to the literally identical pathophysiology hinting at both to be two variants of the same disease, we have recently suggested the uniform use of the term *PNV* for both entities, establishing a new pachychoroid disorder classification system discriminating between PNV with (stage IIIa; overlap with CSC) and PNV without subretinal fluid (stage IIIb)^5^.

Although some recent studies, e.g. Matsumoto *et al*.^6^, have successfully evaluated the effectiveness of photodynamic therapy (PDT) for PNV, many study groups have demonstrated that anti-vascular endothelial growth factor (VEGF) intravitreal therapy is an effective treatment for PNV, and currently only ranibizumab possesses approval by the European Medicines Agency (EMA) for this indication^7–9^. Its effectiveness in PNV has however proven to be variable, which is largely attributable to the still unclear role of subretinal fluid, which might result from both CNV exudation and/or the underlying pachychoroid^8^. As a result, anatomical non-response, defined as persistent fluid, is frequently observed in spite of intense ranibizumab treatment^7,8^.

In a series of clinical and experimental studies, significant differences between ranibizumab and aflibercept have been noted concerning their effects on the choroid and their efficacy in PNV^7,10,11^. The following study was therefore designed to investigate the effects of switching treatment to aflibercept in cases of PNV not adequately responding to previously administered monthly ranibizumab

## Methods

### Participants

For this retrospective cohort study, all eyes presenting with treatment-naïve CNV at the Ludwig Maximilians-University Munich, Germany, between January 2017 and May 2019 were screened for cases of PNV eligible for inclusion in this study, defined as: (i) Presence of a pachychoroid, defined as a subfoveal choroidal thickness ≥350 µm, or ≥200 µm with a previous pre-documented history of CSC (due to choroidal thinning with aging)^12^; (ii) Presence of a CNV; (iii) absence of CNV aneurysms/polyps as defined by the EVEREST I/II study group; (iv) inadequate response to ≥ 3 ranibizumab injections, in spite of monthly dosing, defined as persistence of subretinal fluid four weeks after the last ranibizumab injection; (v) resulting switch to aflibercept administered as three monthly injections; (vi) absence of soft drusen or reticular pseudodrusen on either eye; (vii) absence of anti-mineralocorticoid or photodynamic therapy during follow-up; (viii) absence of confounding comorbidities (diabetic retinopathy, hereditary retinal disease, diseases of the vitreoretinal interface, status after vitrectomy, optic media opacification impeding sufficient image quality). Approval for the experimental protocol of this retrospective chart review was obtained from the Institutional Review Board of the Department of Ophthalmology, Ludwig Maximilian’s University, and the study adhered to the tenets of the Declaration of Helsinki. All patients provided written informed consent.

Epidemiological data was obtained from each patient, including age, gender, previous ocular comorbidities and procedures, date of first diagnosis of PNV or CSC complicated by CNV, date of first ranibizumab injection, number of total ranibizumab injections, date of switching to aflibercept, number of total aflibercept injections, and objective refraction-based Snellen chart visual acuity at baseline and each following visit, which was later converted to logMAR for analysis.

### End points

Primary: Anatomical success rate four weeks after the third aflibercept injection, defined as absence of macular fluid.

Secondary: SFCT, SRF thickness, objective-refraction corrected visual acuity.

### Multimodal imaging

Multimodal imaging (all on Spectralis HRA + OCT, Heidelberg Engineering, Heidelberg, Germany) was performed after pupil dilation with topical tropicamide 1% and phenylephrine 2.5%. It included enhanced depth (EDI) spectral domain optical coherence tomography (SD-OCT) and near-infrared (NIR) confocal laser scanning ophthalmoscopy (CSLO) in every eye at each visit. EDI SD-OCT was acquired using the volume mode in the high-speed setting with 49 B-scans (512 ×496) covering an area of 20° x 20° centered on the macula with the EDI mode “on”. OCT angiography was performed in every eye at baseline. During follow-up, the presence of lessions suggestive of PCV/AT1 was ruled out using OCT, OCT angiography and fluorescein/indocyanine green angiography (FA/ICGA) in all eyes. Blue-autofluorescence (BAF) CSLO and additional OCT angiography scans were performed at the investigator’s discretion.

### Measurement of subretinal fluid (SRF), subfoveal choroidal thickness (SFCT) and central subfield thickness (CST)

Measurements of SRF and SFCT were obtained using the Heidelberg Eye Explorer (Heidelberg Engineering, Heidelberg, Germany) on enhanced depth imaging OCT images in the 1:1 µm setting. SRF thickness was measured at its maximum height from the outer portion of the photoreceptors to the retinal pigment epithelium. SFCT was measured directly underneath the fovea from the outer portion of the retinal pigment epithelium to the sclerochoroidal interface. Automated CST measurements were directly extracted from the software. Where needed, segmentation was manually adjusted.

### Anti-VEGF treatment

All eyes were treated with monthly anti-VEGF injections. Treatment was started on-label with three monthly injections of ranibizumab (Novartis Pharma AG, Basel, Switzerland). A first evaluation of the treatment response was performed four weeks after the third injection. In the case of persistent macular fluid, treatment could be continued with monthly ranibizumab or switched to monthly aflibercept.

### Statistical analysis

All data were gathered and analyzed in Microsoft Excel spreadsheets (Version 16.23 for Mac; Microsoft, Redmond, WA, USA). Statistical analysis was performed in SPSS Statistics 25 (IBM Germany GmbH, Ehningen, Germany). The level to indicate statistical significance was defined as p < 0.05. The Shapiro-Wilk and Kolmogorov-Smirnov tests were employed to test for normal distribution. Statistical analyses of intra-group differences were performed using the dependent two-tailed Student t-test and the Wilcoxon signed rank test. A repeated measures ANOVA test was used to compensate for multiple testing, if applicable. Pearson’s correlation coefficient was used to test associations of dependent and independent variables.

## Results

### Baseline demographics

Fourteen eyes of 14 patients were included in the analysis. Detailed baseline and treatment characteristics can be found in detail in Table 1. In brief, there were 6 right, and 8 left eyes. Mean age was 64.1 ± 7.5 (range: 51–78) years. Female to male ratio was 7 / 7 (50 / 50%). All eyes had a previously documented history of pachychoroid disease, which was chronic CSC in all cases (100%). Previous treatment for CSC was performed in 6 eyes (42.9%), including oral spironolactone (6 eyes, 42.9%), photodynamic therapy (1 eye, 7.1%), subthreshold non-damaging end-point laser therapy (1 eye, 7.1%), and oral acetazolamide (1 eye, 7.1%). Two eyes (14.3%) received multiple treatments, consisting of spironolactone/end-point laser/photodynamic therapy (twice) in one eye (7.1%), and spironolactone/acetazolamide in one eye (7.1%). All previous therapies were administered at least 2 months prior to initiating ranibizumab.

**Table 1:** Baseline demographic and treatment characteristics.

No. of eyes (n)	14
Right/Left	6 / 8
No. of patients (n)	14
Gender (m/f)	7/7
Mean age (years)	64.1 ± 7.5 (range: 51–78)
**Pachychoroid disease stage**	
1 (PPE)	0
2 (CSC)	0
3 (PNV)	14 (100%)
4 (PAT1)	0
Mean Ranibizumab injections until switch (n)	8.4 ± 4.1 (3–15)
Aflibercept injections after switch	3 ± 0 (3–3)
**Mean treatment interval (days)**	30.7 ± 4.1 (28–43)
Ranibizumab treatment interval	30.4 ± 3.9 (28–42)
Aflibercept treatment interval	31.4 ± 4.4 (28–43)

### Macular morphology

At baseline, all eyes (100%) showed subretinal fluid and had a flat, irregular PED forming a double-layer sign. The presence of a type 1 CNV within the flat PED was confirmed on OCT angiography in all eyes (100%). At baseline, no eye showed intraretinal fluid, a serous pigment epithelium detachment or a type 2 CNV configuration. None of the partner eyes showed soft drusen or reticular pseudodrusen/subretinal drusenoid deposits.

On multimodal imaging, no eye was diagnosed with PCV/AT1. At baseline, no eye showed polypoidal/aneurysmal lesions on OCT angiography en-face and B-scans^13^ or OCT features suggestive of PCV/AT1, i.e. pigment epithelial detachment notching, sharply peaked pigment epithelial detachment, or hyperreflective ring^14^. Moreover, no eye showed ICG/FA features typical of PCV/AT1, i.e. polyps/aneurysms, nodular hyperfluorescence, branching vascular networks or interconnecting channels with associated polyps/aneurysms^15,16^.

### Anti-VEGF treatment

All anti-VEGF intravitreal injections were performed monthly in a real-world setting. The mean interval between injections was 30.7 ± 4.1 (28–43) days. There was no difference in the mean dosing interval between ranibizumab (30.4 ± 3.9; 28–42 days) and aflibercept (31.4 ± 4.4; 28–43 days; p = 0.21). All eyes (100%) received at least 3 ranibizumab injections before switching. Switching to aflibercept was performed after mean 8.4 ± 4.1 (3–15) ranibizumab injections. All eyes (100%) received three monthly loading doses of aflibercept after switching. An evaluation of aflibercept outcomes was performed after a mean 35.0 ± 5.3 (28–43) days after the third injection.

### Primary outcome: percentage of eyes with a dry macula

**Figure 1:**
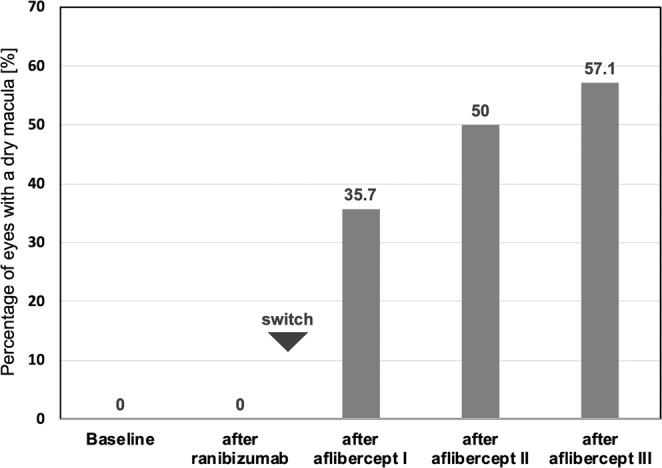
Percentage of eyes with a dry macula. At baseline and after ranibizumab treatment, no eye (0%) showed a dry macula. After the first, second, and third injection of aflibercept, the percentage increased to 35.7, 50.0 and 57.1%.

**Figure 2:**
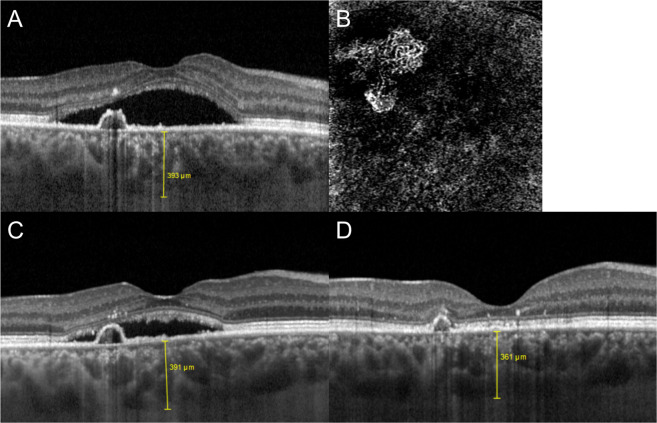
Patient sample demonstrating the efficacy of switching to aflibercept in PNV. A) At baseline, OCT showed marked subretinal fluid with a fibrovascular pigment epithelium detachment in the nasal part of the fovea of this eye with PNV. SFCT was 393 µm. B) On OCT angiography, a type 1 CNV below the RPE could be detected. C) After four injections of ranibizumab, persistence of subretinal fluid as well as an unaltered pigment epithelium detachment could be noted. SFCT remained stable at 391 µm. As a sign of chronicity, shed outer segment debris had started to accumulate just below the elevated photoreceptors in the outer retina. D) After one injection of aflibercept, subretinal fluid was completely eliminated. Moreover, SFCT was reduced to 361 µm, and the pigment epithelium detachment had partially shrunk.

None of the 14 eyes (0%) achieved a dry macula status during the ranibizumab treatment period, as all showed persistent subretinal fluid at every visit. After the third injection of aflibercept, eight eyes (57.1%) achieved a dry macula status without SRF, IRF or serous PED (Fig. 1 and 2). In these eyes, mean 1.7 ± 0.9 (1–3) aflibercept injections were performed prior to complete fluid resolution. One eye (7.1%) achieved a dry macula status after the first injection of aflibercept, however showed recurrent SRF after the second and third injection.

### Secondary outcome: SFCT, SRF thickness, visual acuity

**Figure 3:**
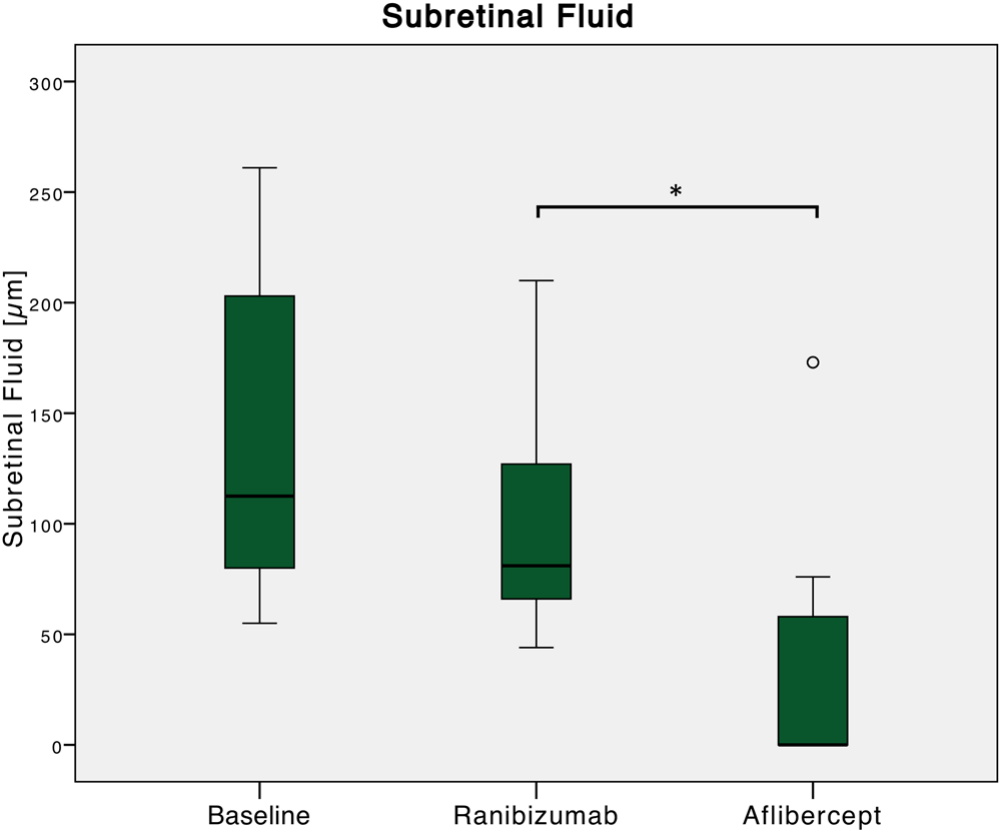
Changes in maximum subretinal fluid (SRF) thickness relative to treatment switching. Before treatment, baseline maximum SRF thickness was 134 ± 66 (55–261) µm. During the ranibizumab treatment period, it did not significantly decrease (before switching: 102 ± 48; 44–210 µm; p = 0.084). After three aflibercept injections, a significant decrease in maximum SRF thickness to 39 ± 62 (0–173) µm was noted (p = 0.0009).

Detailed treatment outcomes are given in Table 2. Before treatment, baseline maximum SRF thickness was 134 ± 66 (55–261) µm. During the ranibizumab treatment period, it did not significantly decrease (before switching: 102 ± 48; 44–210 µm; p = 0.084). After three aflibercept injections, a significant decrease in maximum SRF thickness to 39 ± 62 (0–173) µm was noted (p = 0.0009; Fig. 3).

**Table 2:** Treatment outcomes of switching from ranibizumab to aflibercept.

**Eyes with a dry macula (n)**	
before treatment	0 (0.0%)
after ranibizumab, before switching	0 (0.0%)
after first aflibercept injection	5 (35.7%)
after second aflibercept injection	7 (50.0%)
after third aflibercept injection	8 (57.1%)
**Maximum subretinal fluid thickness (µm)**	
before treatment	134 ± 66 (55–261)
after ranibizumab, before switching	102 ± 48 (44–210)
after first aflibercept injection	49 ± 53 (0–157)
after second aflibercept injection	39 ± 52 (0–164)
after third aflibercept injection	39 ± 62 (0–173)
**Maximum subfoveal choroidal thickness (µm)**	
before treatment	333 ± 99 (274–542)
after ranibizumab, before switching	302 ± 82 (182–514)
after first aflibercept injection	282 ± 89 (137–470)
after second aflibercept injection	269 ± 86 (139–442)
after third aflibercept injection	269 ± 93 (134–447)
**Maximum central subfield thickness (µm)**	
before treatment	372 ± 74 (253–556)
after ranibizumab, before switching	327 ± 78 (252–545)
after first aflibercept injection	285 ± 103 (181–573)
after second aflibercept injection	277 ± 92 (185–532)
after third aflibercept injection	285 ± 98 (203–556)
**Visual acuity (logMAR)**	
before treatment	0.6 ± 0.4 (0.1–1.1)
after ranibizumab, before switching	0.5 ± 0.3 (0.1–1.3)
after first aflibercept injection	0.4 ± 0.3 (0.0–1.3)
after second aflibercept injection	0.5 ± 0.3 (0.0–1.3)
after third aflibercept injection	0.4 ± 0.3 (0.0–1.2)

**Figure 4:**
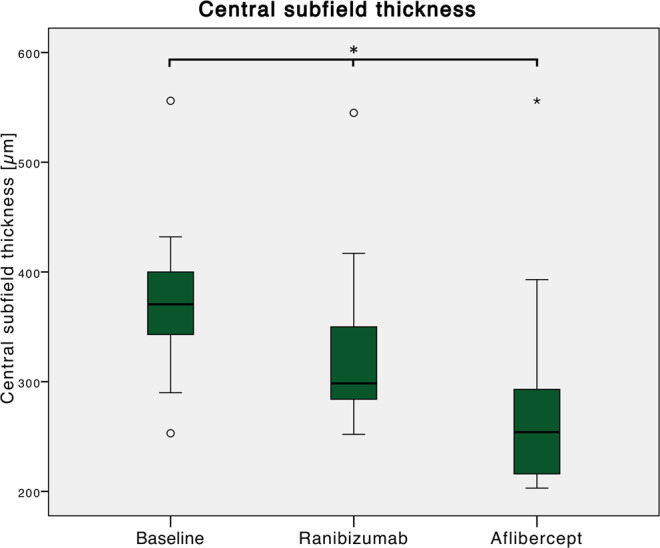
Changes in central subfield thickness (CST) thickness relative to treatment switching. Baseline CST was 372 ± 74 (253–556) µm. During the ranibizumab treatment period, it significantly decreased to 327 ± 78 (252–545 µm) before switching (p = 0.027). After three aflibercept injections, a further decrease to 285 ± 98 (203–556) µm was noted (p = 0.003).

Baseline CST was 372 ± 74 (253–556) µm. During the ranibizumab treatment period, it significantly decreased to 327 ± 78 (252–545 µm) before switching (p = 0.027). After three aflibercept injections, a further decrease to 285 ± 98 (203–556) µm was noted (p = 0.003; Fig. 4).

**Figure 5:**
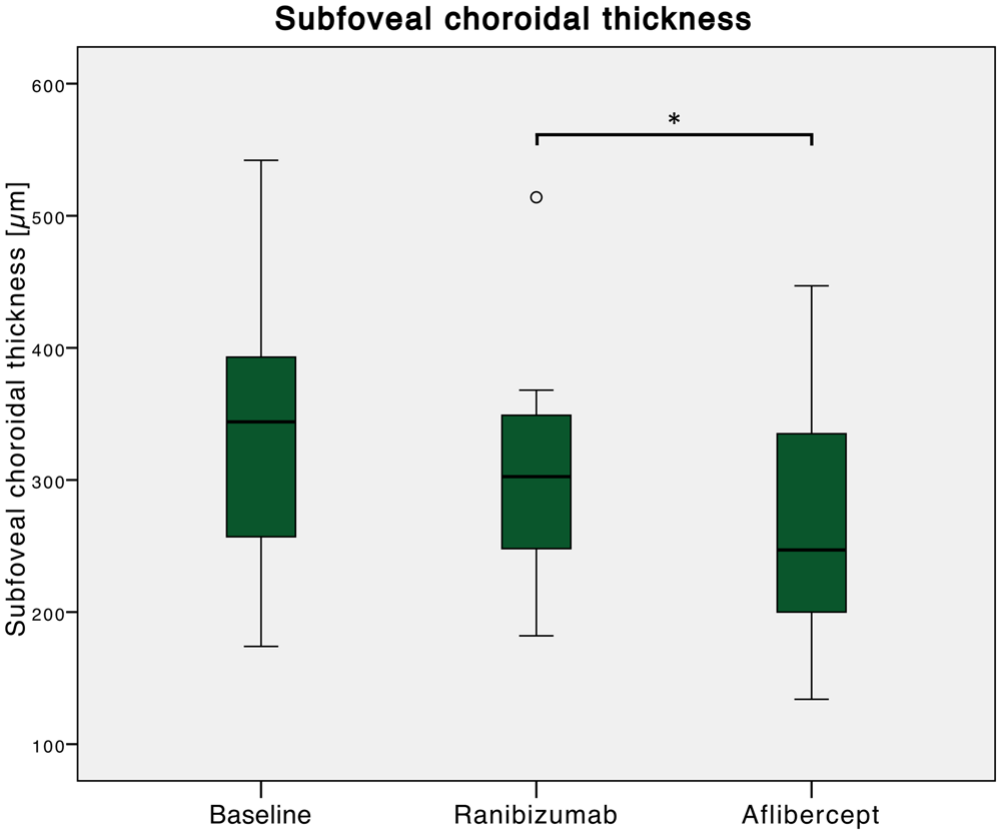
Changes in subfoveal choroidal thickness (SFCT) relative to treatment switching. Baseline SFCT was 333 ± 99 (274–542) µm. During the ranibizumab treatment period, it did not significantly decrease (before switching: 302 ± 82; 182–514 µm; p = 0.13). After three aflibercept injections, a significant decrease in SFCT to 269 ± 93 (134–447) µm was noted (p = 0.044).

Baseline SFCT was 333 ± 99 (274–542) µm. During the ranibizumab treatment period, it did not significantly decrease (before switching: 302 ± 82; 182–514 µm; p = 0.13). After three aflibercept injections, a significant decrease in SFCT to 269 ± 93 (134–447) µm was noted (p = 0.044; Fig. 5).

Baseline objective refraction corrected visual acuity was 0.6 ± 0.4 (0.1–1.1) logMAR. Neither ranibizumab (p = 0.37), nor aflibercept (p = 0.10) treatment led to a significant improvement in visual acuity (after ranibizumab: 0.5 ± 0.3 (0.1–1.3) logMAR; after aflibercept: 0.4 ± 0.3 (0.0–1.2) logMAR).

### Adverse events

No serious adverse events (no endophthalmitis, no retinal detachment, and no macular hemorrhage involving the fovea and requiring pneumatic displacement) were observed during the study period.

## Discussion

The present study was conducted to test the short-term response of intravitreal aflibercept in the treatment of PNV not adequately responding to intravitreal ranibizumab, defined as persistent macular fluid after ≥ 3 ranibizumab injections. According to recent data, such non-response in PNV might affect up to 50% of patients^7^.

In spite of a previous treatment with mean 8.4 ranibizumab injections, no eye included in this study showed a dry macula status before switching. In accordance, both subretinal fluid as well as SFCT did not show a significant decrease during ranibizumab treatment. Strikingly, switching treatment to aflibercept administered in three monthly loading doses however led to a significant decrease in sub-retinal fluid and SFCT, resulting in a dry macula status in 57% of eyes, in spite of the prior complete ranibizumab non-response.

In this study, and generally in PNV, the question of disease activity and resulting treatment decisions are mainly based on the presence of subretinal fluid. In contrast to other neovascular diseases associated with CNV, e.g. AMD or myopia, subretinal fluid in pachychoroid disease can however pose a diagnostic conundrum as its presence can equally result from neovascular activity, i.e. CNV exudation, or the pachychoroid disease itself, defined by choroidal hyperpermeability and leakage^2^. At the moment, no imaging modality has been shown to safely differentiate between subretinal fluid originating from CNV or the pachychoroid.

Thus, two reasons could explain why aflibercept showed better fluid resolution than ranibizumab in our study. On the one hand, more probable according to our data, it might be possible that CNV growth and leakage was better addressed by aflibercept. This could be due to its higher binding affinity to VEGF-A, or due to its broader mechanism of action, which also includes Placental Growth Factor (PlGF)^17,18^. Of note, recent data suggest that VEGF might not play such a prominent role in PNV as in neovascular AMD, and thus, other growth factors, e.g. PlGF, might be more involved^19^. Thus, superior CNV inactivation by aflibercept might secondarily induce choroidal thinning, as leakage from the CNV lesion into the choroid, maybe even choroidal stroma, might be reduced. Indeed, Invernizzi *et al*. have shown that choroidal thickness and choroidal vascularity index are highly correlated with activity of CNV in neovascular AMD^20^. In turn, successful CNV inactivation by aflibercept might be more effective in a secondary choroidal thinning, and thus reduce the disease burden more effectively.

On the other hand, choroidal thinning might hypothetically also result from superior anti-VEGF action of aflibercept on the choroid. While both ranibizumab and aflibercept have proven to be effective in the treatment of PNV^7,8,21^, Jung *et al*.^7^ have indeed recently suggested that aflibercept might be more effective on PNV due its stronger effects on the choroid. In their study of 54 PNV eyes, they found that a dry macula status was achieved in 82.6% of aflibercept, and 51.6% of ranibizumab treated eyes after three loading doses, an effect which seemed to be largely explained by choroidal features. Moreover, Kim *et al*. recently found that aflibercept induced more choroidal thinning in eyes with neovascular AMD than ranibizumab; strikingly, this effect was strongest in eyes with an underlying pachychoroid etiology, i.e. polypoidal choroidal vasculopathy/pachychoroid aneurysmal type 1 CNV^10^. As stated above, as a major caveat, choroidal thinning might however also result from CNV or branching vascular network/polyp inactivation. Clinicians should bear in mind that anti-VEGF has not been shown to act successfully on acute CSC and its underlying pachychoroid^22^. Therefore, additional studies are needed to investigate whether response to different anti-VEGF substances in PNV is related to choroidal responses.

In our study, ranibizumab only showed an effect on CST, but not on SRF. As CST incorporates the distance from the ILM to Bruch’s membrane (including retinal thickness, intra-/subretinal fluid and pigment epithelium detachments), this indicates that ranibizumab largely modulated PED height including the CNV, and to a lesser extend SRF, which thus might mostly result from the pachychoroid. Unfortunately, due to the retrospective nature of our study, we lack longitudinal repeated indocyanine green angiograms to prove this hypothesis by analyzing differential effects of both substances on the severity of choroidal leakage. Moreover, future longitudinal analyses of CNV morphology on OCT angiography before and after switching treatment might give better evidence if vascular remodeling (e.g. CNV size) is better achieved by one substance or the other. As an indirect measure, we were however able to show that PNV non-response to ranibizumab, defined as persisting subretinal fluid, was associated with a stable choroidal thickness – which, in other words, did not show any influence of ranibizumab. In contrast, choroidal thickness however significantly decreased after switching to aflibercept - a finding that was in turn associated with a resolution of sub-retinal fluid in more than half of the eyes included. At this moment, we do not know whether this is due to the superiority of aflibercept in CNV inactivation, or its effects on the choroid.

Certain limitations to our study can be found. One lies within the lack of ICG at baseline, which makes the standard assessment of polyps/aneurysms difficult. As a substitute, we however performed an in-depth analysis of typical OCT and OCT angiography to rule out the diagnosis of PCV/AT1; both OCT and OCT-A alone have been shown to produce a negative predictive value of more than 0.95^13,14^. Thus, the presence of undetected polypoidal/aneurysmal lesions at baseline in our cohort cannot be completely ruled out, but seems highly unlikely; as ICG as gold standard was however only performed during follow-up, a very small probability of polyps/aneurysms being present at baseline regressing during anti-VEGF therapy remains. Moreover, our study has a limited sample size, which reduces our statistical power, and the retrospective nature of this report does not allow for a comparison with a control group continued on ranibizumab. As pointed out by Ferris *et al*. in a report on the effects of anti-VEGF treatment switching in neovascular AMD and diabetic macular edema^23^, a significant amount of patients primarily presenting with suboptimal treatment responses in the beginning can show good late anatomical and functional outcome after a prolonged period of treatment, which might falsely increase the number of patients supposedly “finally reacting” to a treatment switch. Regarding our data, we however believe that this aspect might be of lesser importance as the cohort reported in this study received a rather high amount of mean 8 ranibizumab injections prior to switching, and anatomical outcomes were already evaluated after a short follow-up of only three injections of aflibercept. On the other hand, this short follow-up represents a limitation in assessing final visual acuity after switching, as a prolonged therapy with aflibercept might allow for a better reorganization of retinal layers once a dry macula is achieved. Moreover, even if we report anatomical success, no improvement in mean visual acuity could be found – which might be due to the cumulative damage caused by CSC previous to its neovascular conversion to PNV. In this context, sub-retinal fluid might either be secondary in its influence on visual acuity, or other end points beyond visual acuity, e.g. microperimetry might be more suited to estimate the translation of improved anatomy into improved function.

In conclusion, this study demonstrates that switching to aflibercept induces a favorable short-term response in cases of PNV not responding to previous intravitreal ranibizumab. Further prospective randomized clinical trials with a longer follow-up are warranted.
